# Supplementary material for: Determination of optical density (OD) of oligodeoxynucleotide from HPLC peak area
Source: PeerJ Anal Chem. Author manuscript; Available in PMC 2022 Jul 17. (PMC9288849; doi:10.7717/peerj-achem.20)
Supplement: supporting information2 [file NIHMS1822338-supplement-supporting_information2.docx]

Supporting Information for

**Determination of optical density (OD) of oligodeoxynucleotide from HPLC peak area**

Komal Chillar, Yipeng Yin, Dhananjani N. A. M. Eriyagama and Shiyue Fang*

*Department of Chemistry, Michigan Technological University*

*1400 Townsend Drive, Houghton, Michigan 49931, USA*

Email: shifang@mtu.edu

**HPLC profiles of ODNs 1a, 1b and 1c**

RP HPLC of ODN **1a**

(4 µL out of 1 mL solution of ODN from 1 µmol synthesis injected; HPLC peak area is 485.042)

RP HPLC of ODN **1a**

(6 µL out of 1 mL solution of ODN from 1 µmol synthesis injected; HPLC peak area is 781.314)

RP HPLC of ODN **1a**

(9 µL out of 1 mL solution of ODN from 1 µmol synthesis injected; HPLC peak area is 1113.091)

RP HPLC of ODN **1a**

(12 µL out of 1 mL solution of ODN from 1 µmol synthesis injected; HPLC peak area is 1497.636)

RP HPLC of ODN **1a**

(15 µL out of 1 mL solution of ODN from 1 µmol synthesis injected; HPLC peak area is 1925.099)

RP HPLC of ODN **1a**

(20 µL out of 1 mL solution of ODN from 1 µmol synthesis injected; HPLC peak area is 2408.306)

RP HPLC of ODN **1b**

Crude, trityl-tagged

RP HPLC of ODN **1b**

(50 µL out of 200µL solution of ODN from 0.2 µmol synthesis injected; HPLC peak area is 498.294)

RP HPLC of ODN **1c**

Crude, trityl-tagged

RP HPLC of ODN **1c**

(20 µL out of 200 µL solution of ODN from 0.2 µmol synthesis injected; HPLC peak area is 429.959)
