# Supplementary material for: Determination of optical density (OD) of oligodeoxynucleotide from HPLC peak area
Source: PeerJ Anal Chem. Author manuscript; Available in PMC 2022 Jul 17. (PMC9288849; doi:10.7717/peerj-achem.20)
Supplement: supporting information3 [file NIHMS1822338-supplement-supporting_information3.docx]

Supporting Information for

Determination of optical density (OD) of oligodeoxynucleotide from HPLC peak area

Komal Chillar, Yipeng Yin, Dhananjani N. A. M. Eriyagama and Shiyue Fang*

Department of Chemistry, Michigan Technological University

1400 Townsend Drive, Houghton, Michigan 49931, USA

Email: shifang@mtu.edu

**Protocol for establishing a correlation curve between HPLC peak area and OD_260_**

1. Synthesize an ODN (e.g. 20-mer at 1 µmol scale), and purify with HPLC or use any ODN a lab already has.
2. Dissolve in water (e.g. 1 mL).
3. Inject certain volume (e.g. 4 µL) into HPLC.
4. Collect fractions under the ODN peak, and record the peak area.
5. Combine the fractions and evaporate to dryness.
6. Dissolve the ODN in 1 mL water.
7. Transfer the solution into a 1 mL cuvette with a 1 cm light path.
8. Measure UV absorption at 260 nm to obtain OD_260_.
9. Repeat steps 3-8 for different volumes of ODN solution (e.g. 9, 15 and 20 µL).
10. Plot the OD_260_ numbers from step 8 against peak areas from step 4 to obtain the correlation curve (e.g. use Microsoft Excel).
